# Ifosfamide, cisplatin and etoposide combination in locally advanced inoperable non-small-cell lung cancer: a phase II study

**DOI:** 10.1038/sj.bjc.6690803

**Published:** 1999-11

**Authors:** A F Scinto, V Ferraresi, M Milella, E Tucci, C Santomaggio, R Pasquali-Lasagni, M R Del Vecchio, N Campioni, M Nardi, F Cognetti

**Affiliations:** Division of Medical Oncology I, Division of Surgical Oncology III, Department of Radiotherapy, Regina Elena Cancer Institute, Viale Regina Elena 291, Rome 00161, Italy; Department of Radiotherapy, Hospital S Maria dell Scala, Piazza Duomo 2, Siena 53100, Italy; Pneumology Unit, USL 10/D, Viale Pieraccini 24, Florence 50139, Italy

**Keywords:** non-small-cell lung cancer, locally advanced, inoperable, chemotherapy, ICE, G-CSF

## Abstract

From March 1993 to February 1997, 43 eligible patients with inoperable stage IIIA (ten patients) and stage IIIB (33 patients), histologically confirmed NSCLC received 3 courses of the ICE combination (ifosfamide 1.5 g m^−2^ and mesna 750 mg m^−2^ two times a day, cisplatin 25 mg m^−2^ and etoposide 100 mg m^−2^, all administered intravenously (i.v.) on days 1–3 every 3 weeks) with G-CSF support. After three cycles, patients were submitted to radical surgery or received two additional courses of the ICE regimen and/or curative radiotherapy. Grade 3–4 neutropenia occurred in 21% of 114 evaluable courses, but was of short duration, leading to neutropenic fever in 5% of the courses. Severe thrombocytopenia and anaemia were observed in 13% and 3% of the courses respectively. Non-haematological toxicity was generally mild with only two episodes of reversible renal impairment. The overall response rate after three chemotherapy courses was 69% (28 partial responses, one complete response). Ten patients (8/10 patients in stage IIIA, 2/33 patients in stage IIIB) underwent radical surgery. Median TTP for patients not undergoing surgery (*n* = 33) was 8 months (range 3–34+); median DFS for patients rendered NED by surgery (*n* = 10) was 26 months (range 1–54+). Median OS for the entire group was 12.5 months (range 2–57+). The ICE regimen is active in locally advanced NSCLC with acceptable toxicity and warrants further exploration as induction chemotherapy in larger series. © 1999 Cancer Research Campaign


*British Journal of Cancer* (1999) **81**(6), 1031–1036
© 1999 Cancer Research Campaign
Article no. bjoc.1999.0803

### Ifosfamide, cisplatin and etoposide combination in locally advanced inoperable non-small-cell lung cancer: a phase II study

**AF Scinto[1], V Ferraresi[1], M Milella[1], E Tucci[4], C Santomaggio[5], R Pasquali-Lasagni[2], MR Del Vecchio[3], N Campioni[2], M Nardi[1] and F Cognetti[1]**

[1]Division of Medical Oncology I, [2]Division of Surgical Oncology III, [3]Department of Radiotherapy, Regina Elena Cancer Institute, Viale Regina Elena 291, 00161 Rome, Italy; [4]Department of Radiotherapy, Hospital S Maria dell Scala, Piazza Duomo 2, 53100 Siena, Italy; [5]Pneumology Unit, USL 10/D, Viale Pieraccini 24, 50139 Florence, Italy

**Summary** From March 1993 to February 1997, 43 eligible patients with inoperable stage IIIA (ten patients) and stage IIIB (33 patients), histologically confirmed NSCLC received 3 courses of the ICE combination (ifosfamide 1.5 g $m^{-2}$ and mesna 750 mg $m^{-2}$ two times a day, cisplatin 25 mg $m^{-2}$ and etoposide 100 mg $m^{-2}$, all administered intravenously (i.v.) on days 1–3 every 3 weeks) with G-CSF support. After three cycles, patients were submitted to radical surgery or received two additional courses of the ICE regimen and/or curative radiotherapy. Grade 3–4 neutropenia occurred in 21% of 114 evaluable courses, but was of short duration, leading to neutropenic fever in 5% of the courses. Severe thrombocytopenia and anaemia were observed in 13% and 3% of the courses respectively. Non-haematological toxicity was generally mild with only two episodes of reversible renal impairment. The overall response rate after three chemotherapy courses was 69% (28 partial responses, one complete response). Ten patients (8/10 patients in stage IIIA, 2/33 patients in stage IIIB) underwent radical surgery. Median TTP for patients not undergoing surgery ($n$ = 33) was 8 months (range 3–34+); median DFS for patients rendered NED by surgery ($n$ = 10) was 26 months (range 1–54+). Median OS for the entire group was 12.5 months (range 2–57+). The ICE regimen is active in locally advanced NSCLC with acceptable toxicity and warrants further exploration as induction chemotherapy in larger series. © 1999 Cancer Research Campaign

**Keywords**: non-small-cell lung cancer; locally advanced; inoperable; chemotherapy; ICE; G-CSF

Non-small-cell lung cancer (NSCLC), which accounts for 81% of lung cancers (478 000 new cases per year), is the leading cause of death from malignant disease in men in Western countries (28% of all cancer deaths) (Parkin and Saxo, 1993). Pulmonary resection provides high chance for cure in NSCLC patients with early-stage disease, but the majority of patients (70%) have either locally advanced lesions or disseminated disease at diagnosis.

Locally advanced NSCLC is a heterogeneous disease with a 5-year survival ranging from 40% for T3N0–1 disease to 10–30% and less than 10% for N2 and stage IIIB disease respectively. Selected patients with minimal N2 involvement (not clinically apparent mediastinal disease) are considered as candidates for surgery with a 5-year survival rate of 10–20%. However, the majority of patients in stage IIIA presents with clinical N2 disease, which is considered by most surgeons to be inoperable, with few 5-year survivors after surgical resection alone (Ginsberg et al, 1997).

In patients with stage IIIB disease, surgical treatment is not feasible due to the extent of local tumour spread and chest irradiation is considered the standard treatment. However, the median survival of patients treated with radiotherapy alone is only approximately 10 months with a 5-year survival of less than 10%, due to a high rate of both local failure (40–60%) and distant metastases (35–50%) (Choi, 1983; Perez, et al, 1987).

*Received 6 January 1999*
*Revised 27 May 1999*
*Accepted 7 June 1999*

*Correspondence to:* F Cognetti

Meta-analysis results (Non-small Cell Lung Cancer Collaborative Group, 1995) demonstrate a survival benefit with the use of platinum-based chemotherapy in both locally advanced (13% reduction in the risk of death as compared to radical radiotherapy alone) and metastatic disease (27% reduction in the risk of death as compared to best supportive care).

The early use of chemotherapy in the treatment of locally advanced NSCLC might offer the opportunity to treat widespread sub-clinical disease and might enable the resection of some inoperable lesions. In addition, response rate in locally advanced disease is much higher than that achievable in metastatic setting, ranging from 40% to 60%, and combined modality therapy (i.e. a local treatment modality plus an effective systemic therapy) has been demonstrated to improve survival in selected stage III patients (Roth et al, 1994; Bonomi and Penfield, 1997; Rosell et al, 1994). Combination chemotherapy has shown superior activity as compared to single-agents and platinum-based regimens are considered the treatment of choice (Ginsberg et al, 1997).

Among the most active drugs in NSCLC, ifosfamide, as single agent, produces an objective response rate of approximately 25% in metastatic disease and the use of the uroprotective agent mesna permits its safe use in combination with cisplatin (Eberhardt and Niederle, 1992).

In the present study, we have explored the toxicity and activity of a combination regimen including ifosfamide, cisplatin and etoposide (ICE) as initial treatment for locally advanced inoperable NSCLC. Based on previous experiences with the ICE regimen, which had shown myelosuppression to be a significant dose-limiting toxicity (Ardizzoni et al, 1987; Paccagnella et al,

1990; Pujol et al, 1990; Shepherd et al, 1992; Shirinian et al, 1992; Fischer et al, 1994; Perez et al, 1994), the treatment was administered with granulocyte colony-stimulating factor (G-CSF) support.

### PATIENTS AND METHODS

### Eligibility and treatment evaluation

Between March 1993 and February 1997, 44 patients with histologically or cytologically confirmed stage III (according to the tumour-node-metastasis (TNM) classification (American Joint Committee, 1992) NSCLC were entered in this phase II study. Eligibility criteria included: radically unresectable disease (as evaluated by the thoracic surgeon, stages IIIA and IIIB), presence of bidimensionally measurable lesions, performance status (WHO) of 0–2, absolute neutrophil count (ANC) above $1.5 \times 10^9$ $l^{-1}$, platelet count above $100.0 \times 10^9$ $l^{-1}$, adequate renal (creatinine clearance ≥ 60 ml $min^{-1}$) and hepatic function.

Exclusion criteria included: pregnant or lactating women, or women of childbearing potential not using adequate contraception; previous treatments for NSCLC; concomitant serious medical illnesses.

Pretreatment evaluation included: physical examination, complete blood count, platelet count, serum chemistries, creatinine clearance, electrocardiogram (ECG), chest X-ray, computerized tomography (CT) scan of the brain, chest and upper abdomen, and radionuclide bone scan. Whenever the N status was unclear, as evaluated by clinical/radiological studies, mediastinoscopy was performed to better define the stage. Weekly complete blood count and platelet count, serum chemistries, physical examination and chest X-ray were performed before each course of treatment. In the first ten patients, complete blood count and platelet count were performed every other day to better define haematological toxicity. Toxicity was graded according to the standard WHO (Miller et al, 1981) criteria.

Response to treatment was assessed by CT scan after three courses and defined according to the standard WHO criteria. Imaging studies were thoroughly examined by a team of independent reviewers composed by a radiologist, a thoracic surgeon and a medical oncologist.

The study protocol was approved by the institutional Ethical Committee and all patients gave their informed consent.

### Treatment plan

Combination chemotherapy was administered, in out-patient setting, every 3 weeks as follows: ifosfamide 1.5 g $m^{-2}$ $day^{-1}$ as 30 min intravenous (i.v.) infusion on days 1–3, cisplatin 25 mg $m^{-2}$ $day^{-1}$ as i.v. infusion following adequate hydration with at least 2 l normal saline on days 1–3, and etoposide 100 mg $m^{-2}$ $day^{-1}$ as 30 min i.v. infusion on days 1–3. The uroprotector mesna at the dose of 1.5 g $m^{-2}$ $day^{-1}$ as i.v. bolus, in two divided doses, was routinely administered.

Prophylactic r-metHuG-CSF was given at the dose of 5 μg $kg^{-1}$ $day^{-1}$ subcutaneously (s.c.) from day 4 to day 14 in the first ten patients. Based on the results of the haematological toxicity analysis performed in these patients, G-CSF was then routinely administered at the dose of 5 μg $kg^{-1}$ every other day from day 4 to day 14.

A 25% dose reduction was planned in the event of grade 4 myelosuppression. The courses were repeated every 3 weeks, provided that the patients had totally recovered from toxicity (ANC above $1.5 \times 10^9$ $l^{-1}$, platelet count above $100.0 \times 10^9 l^{-1}$, any non-haematological toxicity grade ≤1), with a maximum acceptable delay of 2 weeks.

Ondansetron (8 mg i.v.) and dexamethasone (20 mg i.v.) were routinely given 30 min before cisplatin administration, as anti-emetic medication.

**Table 1** Patient characteristics

| | |
|---|---|
| Entered | 44 |
| Eligible | 43 |
| Median age (range) | 58 (38–76) |
| Sex | |
| Male | 36 |
| Female | 7 |
| Median WHO PS (range) | 0 (0–2) |
| 0 | 23 |
| 1 | 18 |
| 2 | 2 |
| Stage | |
| IIIA | 10 |
| IIIB | 33 |
| Histology | |
| Squamous | 28 |
| Adenocarcinoma | 10 |
| Other | 5 |

### Statistical analysis

Time to progression (TTP) was calculated from the first day of treatment to the time of progression. Overall survival (OS) was calculated from the on-study day to the date of death or last follow-up. Continuous data were summarized as the median and range, and 95% confidence intervals (CI) were calculated where indicated. Response duration and survival curves were calculated according to the Kaplan–Meier method (Kaplan and Meier, 1958).

### RESULTS

### Patient characteristics and response to treatment

From March 1993 to February 1997, 44 patients entered the study. Forty-three patients were eligible, one patient who had a diagnosis of small-cell lung cancer at surgery was considered not eligible. Patient characteristics are described in Table 1.

Thirty-eight patients received at least three cycles of the planned treatment; two patients received only two courses, due to early disease progression (one patient) and to treatment-unrelated death (one patient died of acute myocardial infarction); three patients received only one cycle of chemotherapy, due to early progression.

Forty-two patients were evaluable for response and one patient could not be evaluated because of treatment-unrelated death after the second cycle. The overall response rate (ORR) for the whole group was 69% (95% CI 55–83%), 90% (95% CI 71–100%) for patients with stage IIIA, and 62% (95% CI 45–79%) for patients with stage IIIB respectively (Table 2).

Ten patients (8/10 IIIA, 2/33 IIIB) underwent radical surgery after three courses of chemotherapy, with two patients receiving two additional cycles of chemotherapy and radiotherapy (60 Gy) before the surgical treatment respectively.

 © 1999 Cancer Research Campaign

**Table 2** Response to chemotherapy

| Patient population | Response rate (95% CI) |
|---|---|
| Evaluable ($n$ = 42) | 69% (55–83)<br>CR: 1<br>PR: 28<br>SD: 7<br>PD: 6 |
| Stage IIIA ($n$ = 10) | 90% (71–100)<br>PR: 9<br>SD: 1 |
| Stage IIIB ($n$ = 32) | 62% (45–79)<br>CR: 1<br>PR: 19<br>SD: 6<br>PD: 6 |

According to protocol design, 17 patients not amenable to surgery underwent radiotherapy with curative intent. Among these, three patients achieved further reduction in tumour size with respect to post-chemotherapy evaluation, two had stable disease (SD), eight had progressive disease (PD) and four were not evaluable (NE). Based on the preliminary safety data with the study regimen, a protocol amendment was subsequently introduced, allowing for the administration of further chemotherapy up to a maximum of six cycles. Four patients received one to three additional courses (one achieved further reduction in tumour size, two had SD and one had PD) and two patients received two or three additional cycles of chemotherapy followed by radiotherapy (one SD, one NE).

Median TTP for patients not undergoing surgery ($n$ = 33) was 8 months (range 3–34+). Median disease-free survival for patients rendered NE by surgery ($n$ = 10) was 26 months (range 1–54+).

Median OS for the entire group was 12.5 months (range 2–57+) (Figure 2); median OS for resected and non-resected patients was 31 months (range 3–57+) and 10 months (range 2–36+), respectively (data not shown).

### Haematological toxicity

Thirty-nine patients and 114 cycles were evaluable for haematological toxicity. Four patients were not evaluable, due to the lack of complete haematological data. Myelosuppression was the main toxicity observed with grade 3–4 neutropenia occurring in 41% of the patients and in 21% of the cycles respectively. Neutropenic fever was observed in 15% of the patients and in 5% of the courses without life-threatening infections. Grade 3–4 thrombocytopenia occurred in 10% of the patients and in 13% of the cycles with no episodes of bleeding. Grade 3 anaemia was observed in 10% of the patients and 3% of the courses (Table 3). One-week treatment delay was necessary in only 4/101 courses and ifosfamide dose was reduced by 25% in 3/39 patients and by 50% in only two patients, due to haematological toxicity. Median delivered dose-intensity (DI) for each drug was: ifosfamide 1.5 g $m^{-2}$ $week^{-1}$ (100% of the planned DI, range 0.75–1.5), cisplatin 25 mg $m^{-2}$ $week^{-1}$ (100% of the planned DI, range 18.75–25), and etoposide 100 mg $m^{-2}$ $week^{-1}$ (100% of the planned DI, range 75–100).

G-CSF was administered daily in the first ten patients (Figure 1A). Median neutrophil nadir was $1.350 \times 10^9$ $l^{-1}$ (range 16–8874) and occurred between days 10 and 12. Based on our previous

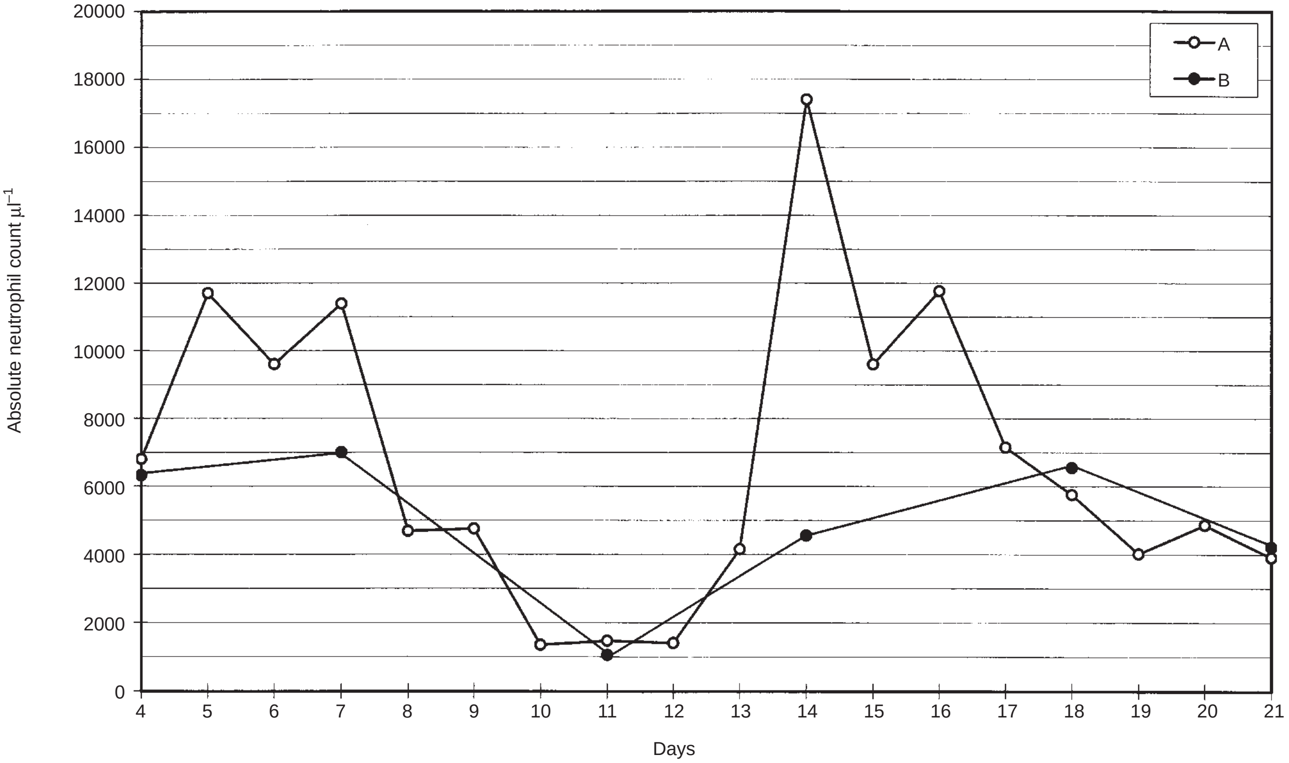

**Figure 1** ANC profile in patients receiving G-CSF supported ICE regimen. The first ten patients received prophylactic G-CSF continuously from day 4 to day 14 (**A**). Subsequent patients ($n$ = 29) received G-CSF on an every other day schedule (**B**). Median ANC profile during the first three courses is shown

© 1999 Cancer Research Campaign

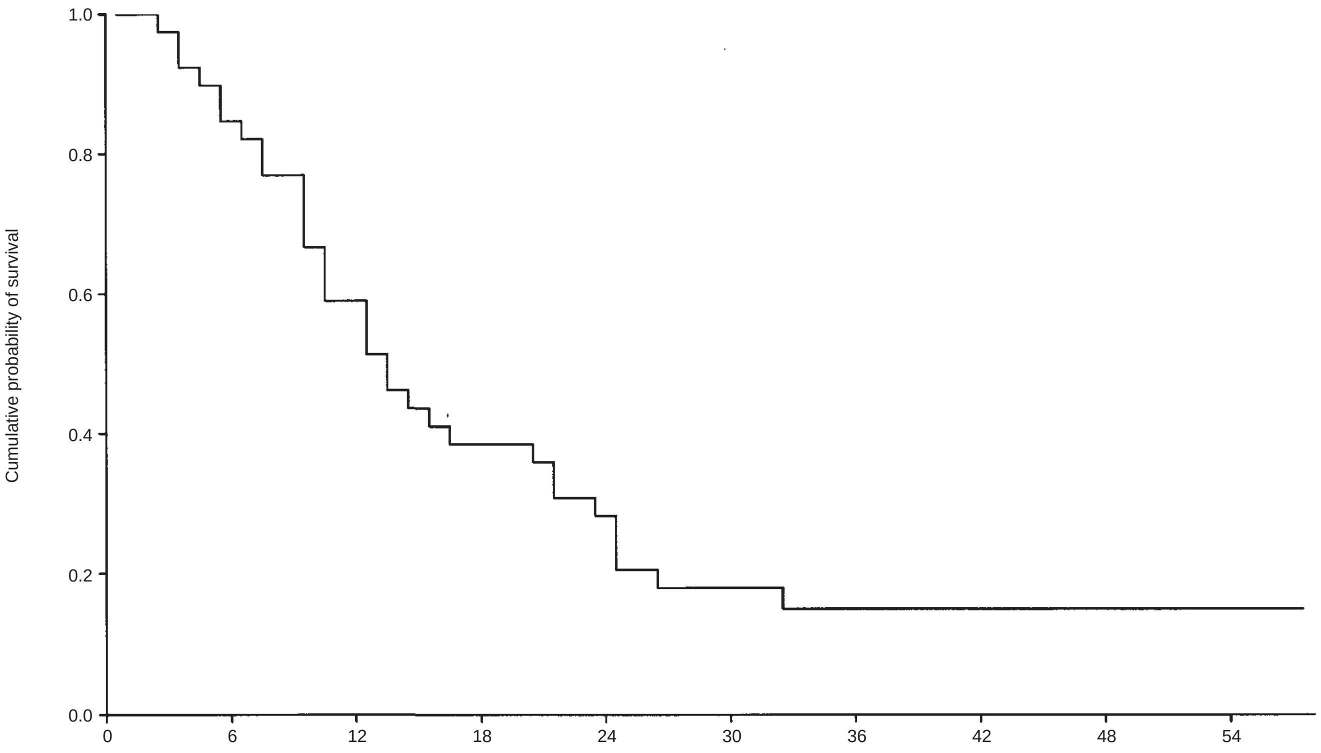

**Figure 2** Overall survival. Kaplan–Meier estimation of cumulative probability of survival. Median OS: 12.5 months (range 2–57+)

experience with different G-CSF schedules to support adjuvant chemotherapy for breast cancer (Calabresi et al, 1995), G-CSF was administered according to an every other day schedule in subsequent patients in order to avoid leucocytosis and due to pharmaco-economic reasons (Figure 1B). Median neutrophil nadir in the latter group was $1.056 \times 10^9$ $l^{-1}$ (range 87–6315). No differences in haematological toxicity were observed among patients treated with daily G-CSF as compared to patients treated with G-CSF administered on every other day.

### Non-haematological toxicity

Alopecia was ubiquitary. A reversible drop in creatinine clearance below 60 ml $min^{-1}$ was observed in only two out of 39 evaluable patients, in the absence of clinical signs and symptoms of renal insufficiency. Both patients had a 1-week treatment delay and one of them also had a 50% cisplatin dose reduction in subsequent cycles. Gastrointestinal toxicity was mild, with grade 3 nausea and vomiting in 13%, grade 1–2 mucositis in 20% and grade 1–2 constipation in 13% of the patients respectively. Eight patients (20%) experienced a mild neurosensorial toxicity. A grade 1–2 increase in liver enzymes was observed in 20% of the patients (Table 4).

### DISCUSSION

This phase II study shows that the ICE combination is an effective regimen in inoperable stage III NSCLC, with an overall response rate of 69% (95% CI 55–83%) and a median overall survival of 12.5 months (range 2–57+). In addition, an impressive resection rate of 80% was observed in the ten patients with stage IIIA disease. Neutropenia was the main toxicity, never leading to life-threatening episodes.

In locally advanced disease first-line chemotherapy achieves a high response rate (range 35–70%), with platinum-containing regimens being considered the treatment of choice. Among the

**Table 3** Haematological toxicity (WHO)

| | Per patient (*n* = 39) | | | | Per cycle (*n* = 114) | | | |
|---|---|---|---|---|---|---|---|---|
| **Toxicity** | **G1** | **G2** | **G3 (%)** | **G4 (%)** | **G1** | **G2** | **G3 (%)** | **G4 (%)** |
| Leucopenia | 5 | 4 | 12 (31) | 5 (13) | 14 | 12 | 15 (13) | 7 (6) |
| Neutropenia | 2 | 3 | 9 (23) | 7 (18) | 6 | 9 | 14 (12) | 10 (9) |
| Neutropenic fever | 2 | 4 | – | – | 2 | 4 | – | – |
| Anaemia | 10 | 15 | 4 (10) | – | 34 | 22 | 4 (3) | – |
| Thrombocytopenia | 7 | 6 | 9 (23) | 1 (3) | 17 | 13 | 14 (12) | 1 (1) |

 *© 1999 Cancer Research Campaign*

**Table 4** Non-haematological toxicity (maximum toxicity per patient, $n$ = 39; WHO)

| | G1 | G2 | G3 (%) | G4 (%) |
|---|---|---|---|---|
| Nausea/vomiting | 10 | 8 | 5 (13) | – |
| Mucositis | 6 | 2 | – | – |
| Renal[a] | 3 | – | – | – |
| Haematuria | 3 | – | – | – |
| Hepatic[b] | 6 | 2 | – | – |
| Stipsis | 4 | 1 | – | – |
| Diarrhoea | – | 1 | – | – |
| Neurotoxicity | 8 | – | – | – |
| Alopecia | – | – | 39 (100) | – |

[a] Renal toxicity includes hyperazotaemia (two patients) and hypercreatininaemia (one patient). Three additional patients had a transient decrease in creatinine clearance (see Results). [b]Hepatic toxicity includes hypertransaminasaemia (G1: one patient; G2: one patient) and increase in alkaline phosphatase levels (G1: five patients; G2: one patient).

several drugs used in association with CDDP, vinca alcaloids and epipodophyllotoxins have proven the most effective (Martini et al, 1993; Bonomi and Penfield, 1997; Elias et al, 1997; Rosell et al, 1997). In spite of a higher toxicity, three-drug combinations seem to be superior in terms of activity and further investigations of new active regimens, with acceptable toxicity, are still needed.

In the last decade, ifosfamide-containing regimens have been investigated by several authors yielding overall response rates ranging from 40% to 70% (Eberhardt and Niederle, 1992) in advanced NSCLC, although significant toxicity, including renal insufficiency, has been reported. In earlier reports of ifosfamide/cisplatin/etoposide regimens, a substantial activity was documented in both locally advanced and metastatic disease. However, severe myelosuppression was the most common dose-limiting toxicity precluding the delivery of the planned DI in a high percentage of patients. In the study by Ardizzoni et al (1987) the projected DI of 1.5 g $m^{-2}$ $week^{-1}$, 30 mg $m^{-2}$ $week^{-1}$, and 60 mg $m^{-2}$ $week^{-1}$ for ifosfamide, cisplatin and etoposide respectively, could not be delivered due to excessive myelotoxicity (two septic deaths and four cases of grade 4 leukopenia out of 13 patients), leading to a reduction in the cisplatin dose to 15 mg $m^{-2}$ $week^{-1}$. In their randomized phase II study, Paccagnella et al (1990) treated 38 patients with ifosfamide (planned DI 1.7 g $m^{-2}$ $week^{-1}$), cisplatin (planned DI 27 mg $m^{-2}$ $week^{-1}$) and etoposide (planned DI 80 mg $m^{-2}$ $week^{-1}$); treatment delays were necessary in 47% of patients, with ten episodes of leukopenia-induced infection and one septic death. In the study by Pujol et al (1990) in 33 patients treated with ifosfamide (planned DI 2 g $m^{-2}$ $week^{-1}$), cisplatin (planned DI 33 mg $m^{-2}$ $week^{-1}$) and etoposide (planned DI 133 mg $m^{-2}$ $week^{-1}$) severe neutropenia was observed in 77% of patients and severe thrombocytopenia in 62%, with one toxic death. Shepherd et al (1992) reported a median neutrophil count at nadir of $0.275 \times 10^9$ $l^{-1}$, with 14 episodes of neutropenic fever and two toxic deaths in 47 patients who received a planned DI of 1 g $m^{-2}$ $week^{-1}$ of ifosfamide, 19 mg $m^{-2}$ $week^{-1}$ of cisplatin and 75 mg $m^{-2}$ $week^{-1}$ of etoposide. Shirinian et al (1992) reported a 90% incidence of severe neutropenia in 20 patients treated with ifosfamide 1.8 mg $m^{-2}$ $day^{-1}$ (days 1–3), cisplatin 20 mg $m^{-2}$ $day^{-1}$ (days 1–3) and etoposide 80 mg $m^{-2}$ $day^{-1}$ (days 1–3) every 3–4 weeks, and a 52% incidence of severe neutropenia in 19 patients treated at lower starting doses (ifosfamide 1.5 mg $m^{-2}$ $day^{-1}$, days 1–3; cisplatin 17 mg $m^{-2}$ $day^{-1}$, days 1–3; etoposide 65 mg $m^{-2}$ $day^{-1}$, days 1–3), with febrile neutropenia with or without documented infection in four patients and one septic death. In the study by Perez et al (1994), 13 patients were treated with ifosfamide (planned DI 0.7 g $m^{-2}$ $week^{-1}$), cisplatin (planned DI 50 mg $m^{-2}$ $week^{-1}$), and etoposide (planned DI 45 mg $m^{-2}$ $week^{-1}$) (dose level –1) with severe leukopenia occurring in 62% of cases and severe thrombocytopenia occurring in 38% of cases; the doses of ifosfamide and etoposide could not be escalated as planned due to severe myelotoxicity (neutropenic fever in two out of two patients treated at dose level 0). In their phase II study, Fischer et al (1994), treated two subsequent cohorts of patients with the ICE combination (ifosfamide 2 g $m^{-2}$ $day^{-1}$, days 1–3; cisplatin 25 mg $m^{-2}$ $day^{-1}$, days 1–3; etoposide 120 mg $m^{-2}$ $day^{-1}$, days 1–3) administered every 4 weeks or every 3 weeks with G-CSF support. In the 14 patients treated at 4-week intervals without G-CSF, a 57% of severe neutropenia was reported, while in the 20 patients treated every 3 weeks with G-CSF support severe neutropenia occurred in 65% of cases, with one episode of neutropenic fever. Interestingly, there was a trend toward a higher response rate in patients receiving intensified chemotherapy with G-CSF support. In the present study, the use of prophylactic G-CSF administration allowed the safe delivery of full doses of chemotherapy (median delivered/planned DI ratio: one for all the three drugs) rarely requiring dose reductions and/or delays. However, despite G-CSF support we observed a relevant incidence of severe haematological toxicity (Table 3), precluding further escalation of ifosfamide doses as originally planned.

Our results with the ICE regimen confirm its high activity in locally advanced patients, although caution should be exerted in evaluating the response rate since the study design did not allow to confirm responses at 4 weeks. The high systemic efficacy of this regimen is also confirmed by the low incidence of non-cerebral distant metastases (4/30 evaluable patients, 13%), with disease progression mainly occurring locoregionally (19/30, 63%) and at brain sites (7/30, 24%) (data not shown).

This observation raises the problem of the optimal strategy for locoregional control in patients not amenable to surgery. Sequential radiotherapy was employed in this study as locoregional treatment in the majority of patients achieving an objective response or a SD after three cycles of chemotherapy, who did not undergo surgery. Although the present study design does not allow to draw firm conclusions about the role of radiotherapy as locoregional treatment after induction chemotherapy, in our subset of patients chest irradiation caused a further reduction in tumour size, with respect to that obtained after chemotherapy, in only 3/19 (16%) patients, while 8/19 patients (42%) experienced PD shortly after the completion of radiotherapy (data not shown). These results are in agreement with those recently reported from a randomized study comparing further chemotherapy to locoregional irradiation in non-metastatic unresectable NSCLC responding to initial chemotherapy (Sculier, 1998).

In stage IIIA-N2, several phase II studies have shown an overall response rate ranging from 30 to 77%, with a pCR rate of 0–14% and a median survival of 11–28 months. A high resection rate of up to 87% has been reported in these studies, although the complete resection rate, when reported, is somewhat lower. The design of phase II studies does not allow to evaluate the benefit of preoperative chemotherapy in terms of survival. Nevertheless, two recently reported small phase III trials indicate that, even in the presence of a limited activity of the induction regimen, survival is significantly higher for chemotherapy-treated patients (Rosell et al, 1994; Roth et al, 1994).

© 1999 Cancer Research Campaign 

In the present study, a very small sample of ten stage IIIA patients was included, all of whom were not suitable for surgical resection at entry. In these patients the ICE combination allowed a complete resection in eight out of ten patients; in addition, two out of 33 stage IIIB patients could be completely resected after induction chemotherapy. A very interesting median disease-free and overall survival of 26 months (range 1–54+) and 31 months (range 3–57+) respectively, was observed in this subgroup of radically resected patients.

In conclusion, the ICE combination is an active regimen in locally advanced NSCLC; with the use of G-CSF support, toxicity was manageable even in out-patient setting. The interesting results in stage IIIA patients warrant further explorations of this regimen as induction chemotherapy in larger series.

### ACKNOWLEDGEMENT

The authors are indebted to Dr Edith A Perez for her helpful comments and critical discussion of the work.

### REFERENCES

American Joint Committee on Cancer (1992) *Manual for Staging of Cancer*, 4th edn, Beahrs OH, Henson DE, Hutter RVP and Kennedy BJ (eds). Lippincott: Philadelphia

Ardizzoni A, Fusco V, Gulisano M, Pronzato P, Bracco F, Capaccio A, Pastorino G, Nosenzo M, Felletti R, Fabiano F and Rosso R (1987) Etoposide, ifosfamide, and cisplatin in the treatment of advanced non-small cell lung cancer. *Cancer Treat Rep* **71**: 1311–1312

Bonomi P and Penfield FL (1997) Postoperative and preoperative chemotherapy for non-small-cell lung cancer. *Cancer Control: J Moffitt Cancer Center* **4**: 297–306

Calabresi F, Papaldo P, Marolla P, Lopez M, Vici P, Di Lauro L, Pellegrini P, Foggi CM, Lepidini G, Antimi M, Terzoli E and Barone C (1995) Different schedules of G-CSF in adjuvant breast cancer therapy with high-dose epirubicin + cyclophosphamide ± lonidamine. *Proc Am Soc Clin Oncol* **14**: 698 (abstract)

Choi NC (1983) Curative radiation therapy for unresectable non-small-cell carcinoma of the lung: indications, techniques, results. In: *Thoracic Oncology*, Choi NC and Grillo HC (eds), pp. 163–199. Raven: New York

Eberhardt W and Niederle N (1992) Ifosfamide in non-small cell lung cancer: a review. *Semin Oncol* **19**: 40–48

Elias AD, Skarin AT, Leong T, Mentzer S, Strauss G, Lynch T, Shulman L, Jacobs C, Abner A, Baldini EH, Frei E III and Sugarbaker DJ (1997) Neoadjuvant therapy for surgically staged IIIA N2 non-small cell lung cancer. *Lung Cancer* **17**: 147–161

Fischer JR, Manegold C, Bülzebruck H, Vogt-Moykopf I and Drings P (1994) Induction chemotherapy with or without recombinant human granulocyte colony-stimulating factor support in locally advanced stage IIIA/B non-small cell lung cancer. *Semin Oncol* **21**: 20–27

Ginsberg RJ, Vokes EE and Raben A (1997) Non-small cell lung cancer. In: *Cancer: Principles and Practice of Oncology*, De Vita VT Jr, Hellman S and Rosenberg SA (eds), pp. 858–911. JB Lippincott: Philadelphia

Kaplan E and Meier P (1958) Nonparametric estimation from incomplete observation. *J Am Stat Assoc* **53**: 457–471

Martini N, Kris MG, Flehinger BJ, Gralla RJ, Bains MS, Burt ME, Heelan R, McCormack PM, Pisters KM and Rigas JR (1993) Preoperative chemotherapy for stage IIIA (N2) lung cancer: the Sloan Kettering experience with 136 patients. *Ann Thorac Surg* **55**: 1365–1374

Miller AB, Hoogstraten B, Staquet M and Winkler A (1981) Reporting results of cancer treatment. *Cancer* **47**: 207–214

Non-small Cell Lung Cancer Collaborative Group (1995) Chemotherapy in non-small cell lung cancer: a meta-analysis using updated data on individual patients from 52 randomized clinical trials. *Br Med J* **311**: 899–909

Paccagnella A, Favaretto A, Brandes A, Ghiotto C, Fornasiero L, Volpi A, Pappagallo G, Festi G, Cipriani, Vinante O, Chiaron-Sileni and Fiorentino MV (1990) Cisplatin, etoposide, and ifosfamide in non-small cell lung carcinoma. *Cancer* **65**: 2631–2634

Parkin DM and Saxo AJ (1993) Lung cancer: worldwide variation in occurrence and proportion attributable to tobacco use. *Lung Cancer* **9**: 1–16

Perez CA, Pajak TF, Rubin P, Simpson JR, Mohiuddin M, Brady LW, Perez-Tamayo R and Rotman M (1987) Long term observations of the patterns of failure in patients with unresectable non-oat cell carcinoma of the lung treated with definitive radiotherapy: Report by the Radiation Therapy Oncology Group. *Cancer* **59**: 1874–1881

Perez EA, Sowray PC, Gardner SL and Gandara DR (1994) Phase I study of high-dose cisplatin, ifosfamide, and etoposide. *Cancer Chemother Pharmacol* **34**: 331–334

Pujol JL, Rossi JF, Le Chevalier T, Daures JP, Rouanet P, Douillard JY, Dubois JB, Arriagada R, Mary H and Godard P (1990) Pilot study of neoadjuvant ifosfamide, cisplatin and etoposide in locally advanced non-small cell lung cancer. *Eur J Cancer Clin Oncol* **26**: 798–801

Rosell R, Gòmez-Codina J, Camps C, Maestre J, Padilla J, Cantò A, Mate JL, Li S, Roig J, Olazàbal A, Canela M, Ariza A, Skachel Z, Morera-Pratt J and Abad A (1994) A randomized trial comparing preoperative chemotherapy plus surgery with surgery alone in patients with non-small-cell lung cancer. *N Engl J Med* **330**: 153–158.

Rosell R, Lòpez-Cabrerizo MP and Astudillo J (1997) Preoperative chemotherapy for stage IIIA non-small cell lung cancer. *Curr Opin Oncol* **9**: 149–155

Roth JA, Fossella F, Komaki R, Ryan B, Putnam JB Jr, Lee JS, Dhingra H, De Caro L, Chasen M, McGavran M, Atkinson EN and Hong WK (1994) A randomized trial comparing perioperative chemotherapy and surgery with surgery alone in resectable stage IIIA non-small-cell lung cancer. *J Natl Cancer Inst* **86**: 673–680

Sculier JP, Paesmans M, Bureau G, Recloux P, Baumöhl J, Van Cutsem O, Berchier MC, Thiriaux J, Lafitte JJ, Mommen P, Van Houtte P, Ninane V and Klastersky J (1998) A phase III randomized study comparing, in non metastatic unresectable non-small cell lung cancer (NSCLC) responding to chemotherapy (CT), further CT to loco-regional irradiation (RT). *Ann Oncol* **9**: 83 (abstr 399)

Shepherd FA, Evans WK, Goss PE, Latreille J, Logan D, Maroun J, Stewart D, Warner E and Paul K (1992) Ifosfamide, cisplatin, and etoposide (ICE) in the treatment of advanced non-small cell lung cancer. *Semin. Oncol* **19**: 54–58

Shirinian M, Lee JS, Dhingra HH, Greenberg J and Hong WK (1992) Phase II study of cisplatin, ifosfamide, and etoposide combination for advanced non-small cell lung cancer: final report. *Semin Oncol* **19**: 58–64

*© 1999 Cancer Research Campaign*
